# Impact of Hypoxia-Induced miR-210 on Pancreatic Cancer

**DOI:** 10.3390/cimb45120611

**Published:** 2023-12-05

**Authors:** Mutian Lian, Maria Mortoglou, Pinar Uysal-Onganer

**Affiliations:** Cancer Mechanisms and Biomarkers Research Group, School of Life Sciences, University of Westminster, London W1W 6UW, UK; w1917186@my.westminster.ac.uk (M.L.); m.mortoglou@westminster.ac.uk (M.M.)

**Keywords:** pancreatic cancer, hypoxia, HIF-1α, microRNA-210

## Abstract

Pancreatic cancer (PC) poses significant clinical challenges, with late-stage diagnosis and limited therapeutic options contributing to its dismal prognosis. A hallmark feature of PC is the presence of a profoundly hypoxic tumour microenvironment, resulting from various factors such as fibrotic stroma, rapid tumour cell proliferation, and poor vascularization. Hypoxia plays a crucial role in promoting aggressive cancer behaviour, therapeutic resistance, and immunosuppression. Previous studies have explored the molecular mechanisms behind hypoxia-induced changes in PC, focusing on the role of hypoxia-inducible factors (HIFs). Among the myriad of molecules affected by hypoxia, microRNA-210 (miR-210) emerges as a central player. It is highly responsive to hypoxia and regulated by HIF-dependent and HIF-independent pathways. miR-210 influences critical cellular processes, including angiogenesis, metastasis, and apoptosis, all of which contribute to PC progression and resistance to treatment. Understanding these pathways provides insights into potential therapeutic targets. Furthermore, investigating the role of miR-210 and its regulation in hypoxia sheds light on the potential development of early diagnostic strategies, which are urgently needed to improve outcomes for PC patients. This review delves into the complexities of PC and introduces the roles of hypoxia and miR-210 in the progression of PC.

## 1. Introduction

Pancreatic cancer (PC) is an aggressive disease with a survival rate of less than 1% after 10 years and 3% after 5 years [1]. PC is often diagnosed at an advanced stage with liver metastasis, making surgical resections challenging due to encroachment on vital arteries and para-aortic metastasis. Symptoms such as abdominal pain and digestive issues, which are present in 70% of PC patients, become more pronounced in later stages [2,3,4]. However, a reliable early detection approach has not yet been developed for PC diagnosis. Currently, there is no reliable biomarker for PC diagnosis. As mentioned above, PC poses a considerable challenge in terms of early diagnosis, as most cases are already metastatic upon initial detection, leaving only a small fraction (9.7%) in a localized stage at diagnosis [5]. These discouraging survival rates have remained relatively unchanged for nearly four decades. Currently, adjuvant chemotherapies like modified-FOLFIRINOX and gemcitabine have demonstrated a longer overall survival rate only by few weeks in PC patients [6]. Even though surgical resection stands as the sole curative option for PC, due to delayed presentation, approximately 15% to 20% of patients qualify for a pancreatectomy [5]. The five-year survival rate after pancreaticoduodenectomy, commonly known as the Whipple procedure, is approximately 21% for cases with negative margin resections and 11% for those with microscopically positive margin resections [7]. Alarmingly, even among patients who undergo negative margin resections with the intention of achieving a cure, up to 71% experience recurrence [7]. This challenge marks the urgent need for a reliable early diagnostic approach to detect PC before it progresses into advanced stages.

The pancreatic ductal adenocarcinoma (PDAC) subtype constitutes most exocrine tumours, encompassing over 90% of all pancreatic malignancies [8]. PDAC originate from epithelial cells lining the pancreatic duct, exhibiting glandular features due to their cellular origin. PDAC often metastasizes, particularly to the liver or lymph nodes [8]. Notably, early PDAC diagnosis is challenging, frequently being detected at an advanced stage, sometimes after metastasis has already occurred [8,9]. Consequently, anti-cancer treatments tend to have limited efficacy due to the cancer’s robust cytoprotective mechanisms that foster drug resistance. Given the aggressiveness and drug resistance of PDAC, the estimated median survival can be less than 4 months [10].

**Figure 1:**
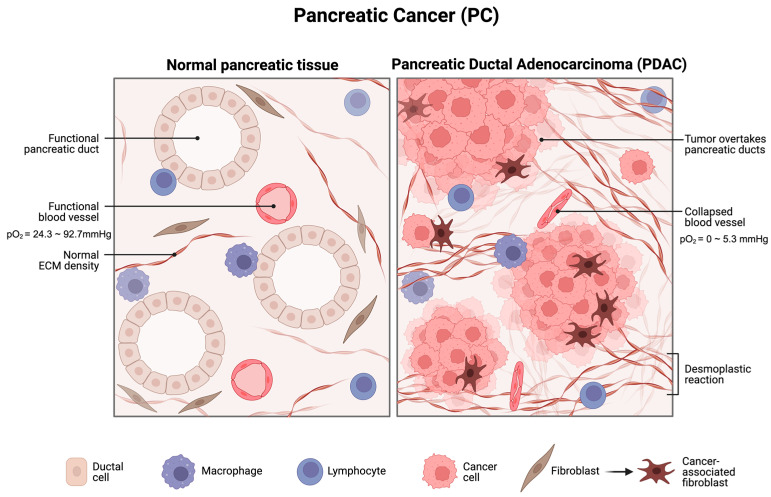
A schematic diagram for PC under profound hypoxia. PDAC displays intensive desmoplastic reaction, proliferation of PC cells, and, more importantly, collapsed blood vessels that significantly lower the oxygen pressure to 0 to 5.3 mmHg compared to 24.3 to 92.7 mmHg in normal pancreatic tissue. Created with BioRender.com (accessed on 10 November 2023).

In comparison to well-oxygenated tumours like thyroid cancer and testicular cancer, PC is frequently distinguished by the presence of profound hypoxic regions, characterized by a median tumour tissue pO_2_ ranging from 0 to 5.3 mmHg (0–0.7%) [11,12]. In stark contrast, adjacent normal pancreatic tissue exhibits significantly higher pO_2_ levels, ranging from 24.3 to 92.7 mmHg (3.2–12.3%) [13,14]. This hypoxic microenvironment within PC arises from factors such as the presence of desmoplastic fibrotic stroma, the rapid proliferation of pancreatic cancer cells, the metabolic shift from phosphorylation to glycolysis, and inadequate vascularization, all of which collectively elevate oxygen consumption while compromising the supply of oxygen [14,15,16] (Figure 1). Notably, the presence of hypoxic areas within PC is strongly associated with tumour progression and suggests an unfavourable prognosis compared to well-oxygenated tumours. It is considered one of the independent prognostic factors for PC [15,17]. This hypoxic microenvironment also exerts a significant influence on various malignant traits of PC, such as metabolic changes, the emergence of cancer stem cells (CSCs), increased invasion and metastasis, and angiogenesis. These factors work in concert to promote the progression and resistance to therapy for PC. Intra-tumoral hypoxia, driven by mechanisms that include maintaining redox balance, triggering autophagy, epigenetic modifications, and responses mediated by hypoxia-inducible factors (HIFs), plays a pivotal role in orchestrating these biological processes in pancreatic cancer [14]. The adaptive response to hypoxia, primarily orchestrated by the HIFs mentioned above, confers a more aggressive and therapeutically resistant phenotype to PC cells [17].

microRNAs (miRNAs/miRs) are short non-coding RNAs that play a pivotal role in post-transcriptional gene regulation by facilitating messenger RNA (mRNA) degradation and impeding translational processes [18]. miRs play a key role in the downregulation of specific target mRNAs through mechanisms that include reducing transcript levels and inhibiting translation. Among the miRs that are influenced by hypoxia via the HIFs-dependent pathway, miR-210 stands out as the most responsive and influential candidate [19]. Previous studies suggest that HIF-1α-mediated upregulation of miR-210 has a direct impact on the alteration of target gene transcription [19]. This alteration is closely associated with disruptions in the cell cycle, insufficient energy production, cell death, and irregular control over cell shape, orientation, and guided movement. These changes are hallmark features of cancer pathology and metastasis [19,20].

In this review, we aim to elucidate the roles of hypoxia-induced miR-210 in PC progression, chemoresistance, diagnosis, and prognosis and, more importantly, emphasize the future direction in utilizing miR-210 expression as a diagnostic marker for early PC detection.

## 2. Mechanisms of Hypoxia-Induced Upregulation of miR-210

### 2.1. Hypoxia

Hypoxia, a condition in which the oxygen levels drop below their normal physiological range, emerges within the progressing tumour and plays a crucial role in shaping the tumour microenvironment (TME). Numerous studies have demonstrated its significant impact on various cellular processes, including angiogenesis, the transition of epithelial cells into mesenchymal cells (EMT), and altering cellular characteristics like acquiring stem cell-like properties. These effects have profound implications for the performance and efficacy of anti-cancer treatments [21]. Reduced oxygen levels promote tumour proliferation and the emergence of immunosuppressive conditions in cancer. Hypoxia triggers a transition within the tumour, shifting it from a benign state to an aggressive one, concurrently stimulating angiogenesis and the acquisition of pathological traits. This latter phenomenon plays a pivotal role in tumour progression. Tumour angiogenesis, marked by its inability to restore physiological pO_2_ levels, not only facilitates tumour cell growth and dissemination but also contributes to immunosuppression and the desensitisation of cytotoxic immune competent cells directed against the tumour [22]. In addition, tumour cellular survival in hypoxic conditions necessitates the initiation of several molecular signalling pathways that promote angiogenesis, stimulate erythropoietin production, and induce metabolic shifts favouring glycolysis. This collective biological reaction is referred to as the hypoxic adaptive response [23].

### 2.2. Roles of HIFs during Hypoxic Adaptive Response

A family of transcription factors activates hypoxic adaptive response called HIFs, whose expression is activated by insufficient local oxygen availability for cell survival through several molecular pathways (Table 1) [24]. The rapid growth of tumour cells elevates oxygen consumption during tumourigenesis, consequently giving rise to an intra-tumoral oxygen gradient due to partial oxygen pressure [25]. The predominant hypoxic responses are orchestrated through three HIF members: HIF-1, HIF-2, and HIF-3 [16,21,24]. These heterodimeric HIFs consist of an oxygen-sensitive α-subunit paired with a β-subunit [24,26]. Under normoxic conditions, the heterodimer remains in a dissociated state. Prolyl hydroxylases (PHDs) catalyse the hydroxylation of two specific proline residues (Proline-402 and Proline-564) within the HIF-1α subunit, using oxygen as a substrate [27]. This hydroxylation event is followed by the ubiquitination of HIF-1α by the tumour suppressor protein, von-Hippel–Lindau protein (pVHL), leading to subsequent proteasomal degradation of the HIF-1α subunit [28]. Conversely, under hypoxic conditions, the activity of PHDs is impaired, rendering them incapable of hydroxylating the HIF-1α subunit. As a result, pVHL is unable to ubiquitinate its target, resulting in the stabilisation of the HIF-1α [16,26]. HIF-2α shares a similar regulation of gene expression with HIF-1α, despite the fact that HIF-1α is widely expressed in vivo, while HIF-2α is predominantly expressed in hepatocytes, glial cells, cardiomyocytes, interstitial cells, kidney fibroblast, and endothelial cells [29,30].

For the structure of the HIF heterodimers, both the α and β subunits share a common structural framework at their amino-terminal ends, featuring basic helix–loop–helix (bHLH) and PAS (PER–ARNT–SIM) domains. These domains play pivotal roles in facilitating heterodimerisation and DNA binding processes [24]. Distinctively, the carboxy-terminal domain of HIF-1α and HIF-2α encompasses specialised domains responsible for governing their stability, notably the oxygen-dependent degradation domain (ODD), as well as domains regulating their transcriptional activity, specifically comprising two transactivation domains (TADs), namely the N-TAD and C-TAD [31].

Moreover, molecular targets of HIF-1 and HIF-2 are predominantly different but partially overlapped. HIF-1 targets endothelial nitric-oxide synthase (NOS3) to maintain endothelial homeostasis [32], monocarboxylate transporter 4 (MCT4) and carbonic anhydrase (CA-IX) to modulate pH level [33], targets phosphofructokinase (PFK) and lactate dehydrogenase A (LDHA) to regulate glycolysis [34], glucose transporter 1 and 3 (GLUT1/3) to regulate metabolism [33], heme oxygenase-1 (HMOX1) to facilitate pro-angiogenic activity [35], and induces apoptosis through targeting BCL2/adenovirus E1B 19 kDa-interacting protein 3 (BNIP3) and BCL2/adenovirus E1B 19 kDa-interacting protein 3-like (BNIP3L/NIX) [33]. HIF-1 also regulates cell survival and proliferation by targeting insulin-like growth factor 2 (IGF2), insulin-like growth factor binding protein 1 (IGF-BP1), IGF-BP3, and c-Myc [33,36]. In contrast, HIF-2 targets matrix metalloproteinases (MMP) 2 and 13, and the stem cell factor OCT-3/4 to regulate blood vessel remodeling, apoptosis, and self-renewal [33]. Shared targets of HIF-1 and HIF-2 encompass vascular endothelial growth factor A (VEGFA), GLUT1, and erythropoietin (EPO) [37]. Nevertheless, it is noteworthy that EPO is primarily regarded as a target gene regulated by HIF-2. Erythropoiesis, a process essential to the production of red blood cells, hinges on the availability of iron. HIFs play a pivotal role in regulating the expression of various genes involved in iron homeostasis, including transferrin [38]. Of particular significance, a conserved iron response element (IRE) identified in the 5′ untranslated region (UTR) of HIF-2α mRNA has been demonstrated to facilitate the translation of HIF-2α protein when iron levels are elevated. This finding establishes a functional connection between hypoxia-related erythropoiesis and the intricate regulation of iron balance [39].

HIF-3α stands apart from HIF-1α and HIF-2α due to disparities in both protein structure and the regulation of gene expression. Historically, HIF-3α has been regarded as an inhibitory factor for HIF-regulated genes. Functionally, HIF-3α operates as a transcriptional regulator, exerting negative feedback on gene expression by engaging in competition with HIF-1α and HIF-2α to bind to transcriptional elements within target genes during periods of hypoxia [40]. It is proposed that HIF-3α engages in a competitive interaction with HIF-1α and HIF-2α for binding to HIF-1β subunits. These competitive bindings result in a reduction in HIF-1 and HIF-2 levels, ultimately leading to the inhibition of the upregulation of target genes associated with HIF-1 and HIF-2 [41]. The expression patterns of HIF-1α and HIF-2α have been extensively studied and documented, while the expression patterns of HIF-3α variants still require more investigation [40]. HIF-3α is expressed in human kidney tissue and lung epithelial epithelium [42].

### 2.3. HIF-Dependent Upregulation of miR-210 during Hypoxia

miR-210, a pivotal miR with significant implications in the pathophysiology of human disorders, is encoded by the MIR210 gene situated on chromosome 11p15.5. The stem-loop structure of this miR is located within an intron of the AK123483 noncoding RNA [43]. This miR has gained prominent recognition as a hypoxamir (hypoxia associated miR), exhibiting increased expression under hypoxic conditions across various primary and transformed cells [44]. Its induction is primarily orchestrated by HIF-1α [45], and miR-210 plays a multifaceted role in numerous cellular and developmental processes. Notably, within the promoter region of miR-210, a hypoxia-responsive element (HRE) has been identified, to which HIF-1α binds directly [43,45]. Additionally, an evolutionarily conserved NF-κB binding site has been identified upstream of the miR-210 stem-loop [43,46], underscoring the importance of HIF-1α in modulating miR-210 expression. In normoxic conditions, endogenous levels of miR-210 remain exceedingly low [44,47]. However, a positive feedback loop driven by HIF-1α binding to the HRE on the proximal promoter of miR-210 leads to the stabilisation of the HIF-1α [19,48,49,50,51]. This effect is further amplified by an increase in the nascent primary transcript, pri-miR-210 [19]. miR-210 can additionally enhance the stability of HIF-1α by targeting the expression of glycerol-3-phosphate dehydrogenase-like 1 (GPD1L), an enzyme responsible for hydroxylating HIFα [51]. Moreover, experimental evidence indicates that the miR-210 feedback loop operates through the targeting and subsequent downregulation of Succinate Dehydrogenase Complex Subunit D (SDHD), which is another inhibitor of HIF-1α, by repressing SDHD. It thus facilitates HIF-1α stabilisation, subsequently amplifying miR-210 production and reinforcing the positive feedback loop [19,52]. Consequently, elevated levels of miR-210 in PDAC tissues have emerged as a predictive marker for tumour hypoxia [52].

### 2.4. HIF-Independent Upregulation of miR-210 during Hypoxia

Hypoxia-induced upregulation of miR-210 also exists in HIF-independent pathways involving p53 and NF-κB [47]. A report suggests that in HIF-β knockout mouse embryonic fibroblasts (MEFs) under hypoxic conditions, accumulation of p53 is observed, which directly triggers the expression of miR-210 [47]. Furthermore, p53-induced miR-210 expression serves as a protective mechanism for cardiomyocytes exposed to hypoxic stress. However, it is worth noting that substantial induction of miR-210 by p53 in this study necessitated deep hypoxia. Under extremely low pO_2_ (0.5% O_2_), miR-210 expression surged approximately 25-fold compared to that in normoxic conditions. Conversely, under milder hypoxic conditions (5% O_2_), the induction of miR-210 was significantly lower: approximately 8-fold lower than the changes observed under 0.5% O_2_ extreme hypoxic exposure [47,53].

**Figure 2:**
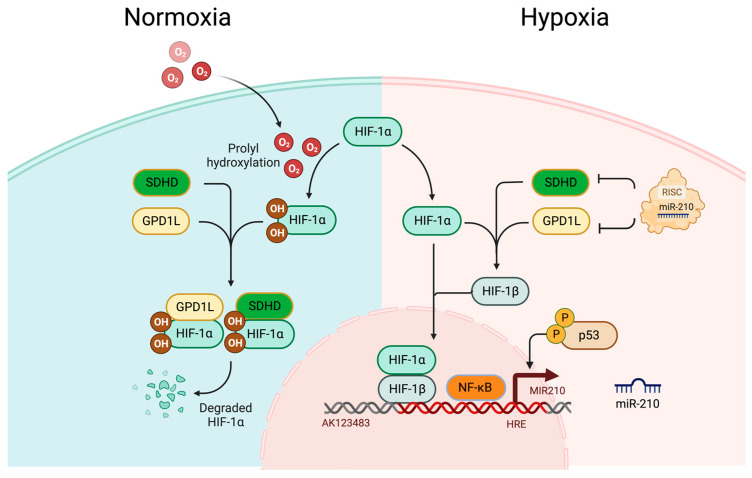
Hypoxia-induced miR-210 upregulation under HIF-dependent pathways and HIF-independent pathways. SDHD stands for Succinate Dehydrogenase Complex Subunit D; GPD1L for glycerol-3-phosphate dehydrogenase-like 1; HRE for Hypoxia Responsive Element. Host gene of miR-210, MIR210 is in the intron region of AK123483 non-coding RNA. Under normoxic conditions, HIF-1α is degraded by interacting with GPD1L and SDHD, while it is stabilised under hypoxic condition by miR-210 through inhibiting GPD1L and SDHD. HIF-independent pathways involve p53 and NF-κB. Hypoxic condition induces accumulation of p53 and NF-κB activation, which triggers miR-210 expression [46,47,53,54]. Created with BioRender.com (accessed on 10 November 2023).

Hypoxic stress also triggers the activation of NF-κB, an extensively studied transcription factor associated with inflammation [47,54]. As mentioned previously, within the miR-210 promoter, there exists a conserved NF-κB binding site located approximately 200 base pairs upstream of the primary stem-loop structure in the pri-miRNA. Evidence like ChIP analyses, promoter–reporter assays, and gene knockdown analyses demonstrates that NF-κB establishes direct interactions with and facilitates the transactivation of the miR-210 promoter specifically under hypoxic conditions [46,47] (Figure 2).

## 3. Roles of miR-210 in Pancreatic Cancer

### 3.1. Hypoxia Induces miR-210 Upregulation in Pancreatic Cancer

Elevated miR-210 expression in PC has been reported frequently associated with hypoxia [55]. Previous research identified that under hypoxic conditions, miR-210 expression was induced in six PC cell lines (AsPC-1, BxPC-3, MiaPaCa-2, PANC-1, Su86.86, and SW1990). Notably, transfection of HIF-1α small interfering RNA (siRNA) a double-stranded, small interference RNA that suppresses HIF-1α expression, into PANC-1 cells led to a significant downregulation of miR-210 expression under hypoxic conditions. miR-210 did not exhibit any discernible impact on the proliferation of PANC-1 or Su86.86 cells. Additionally, dual luciferase reporter assays revealed a significant downregulation of E2F3, EFNA3, GIT2, MNT, ZNF462, and EGR3, indicating potential gene targets by miR-210 [56]. Another study reported an upregulation of miR-210 in diseased tissues compared to control samples [57]. Meanwhile, it was demonstrated that plasma miR-210 expression in patients newly diagnosed with locally advanced PDAC was significantly elevated when compared to age-matched controls [58]. A parallel study showed that serum miR-210-3p holds the potential for distinguishing between patients with PDAC and those with chronic pancreatitis [59]. Ni et al. (2019) also reported that PANC-1 cells exhibited elevated levels of miR-210, HIF-1α, and NF-κB, while the expression of HOXA9 was diminished under hypoxic conditions. HOXA9, a constituent of the homeobox (HOX) family situated within the HOXA cluster, has been identified to be a target of miR-210 in PANC-1 cell line under hypoxic condition. HOXA9 expression is intricately associated with processes such as proliferation, differentiation, and the preservation of progenitor self-renewal [60]. Upon the overexpression of miR-210 in normoxic PANC-1 cells, there was a notable downregulation of epithelial markers E-cadherin and β-catenin, coupled with an upregulation of mesenchymal markers vimentin and N-cadherin, promoting enhanced cell migration and invasive capabilities. Meanwhile, there was a decrease in the expression of HOXA9, and this reduction was correlated with a decrease in sensitivity to the chemotherapeutic drug gemcitabine, an increase in NF-κB expression, and heightened cell migration and invasive abilities. Conversely, the introduction of a miR-210 antagonist into hypoxic PANC-1 cells resulted in an upregulation of E-cadherin and β-catenin levels and a downregulation of Vimentin and N-cadherin levels. This led to reduced cell migration and invasive abilities, alongside an increase in the expression of HOXA9 [61]. Furthermore, an upstream regulator that functions as a competing endogenous RNA (ceRNA) against miR-210 was identified [61]. This firmly established DLEU2L as a ceRNA that interacts with miR-210-3p, highlighting the pivotal role of the DLEU2L/miR-210-3p crosstalk in addressing gemcitabine resistance [61,62]. Moreover, it was proposed that exosomes originating from gemcitabine-resistant PC stem cells play a role in transmitting drug-resistant characteristics to gemcitabine-sensitive pancreatic cancer cells through the delivery of miR-210 [63]. Meanwhile, it was reported that the potent inhibitory impact of metformin, a medication used to treat Type 2 diabetes, on PC cells was further intensified under conditions of low glucose. This enhancement occurred through the suppression of glycolysis and the induction of energy stress, achieved via the upregulation of miR-210-5p [64].

Conversely, it was suggested that miR-210 exhibits lower expression levels in PC tissues compared to adjacent para-cancerous tissues [64]. Moreover, its expression is inversely correlated with both the TNM stage and the tumour size of PC. In vitro experiments revealed downregulation of miR-210 in PC cells in comparison to normal pancreatic ductal epithelial cells. Furthermore, the overexpression of miR-210 leads to cell cycle arrest, reduced cell viability, and downregulates E2F3 expression in PC cells. Dual-Luciferase reporter assays confirm the binding of E2F3 to miR-210. Subsequent experiments provide confirmation that E2F3 is negatively regulated by miR-210 [65]. Therefore, most previous publications agree that hypoxia induces miR-210 upregulation in PC, while there are reports that support the opposite idea that miR-210 is downregulated in PC [65].

### 3.2. Molecular Targets and Hallmarks of miR-210 Upregulation in Pancreatic Cancer

**Figure 3:**
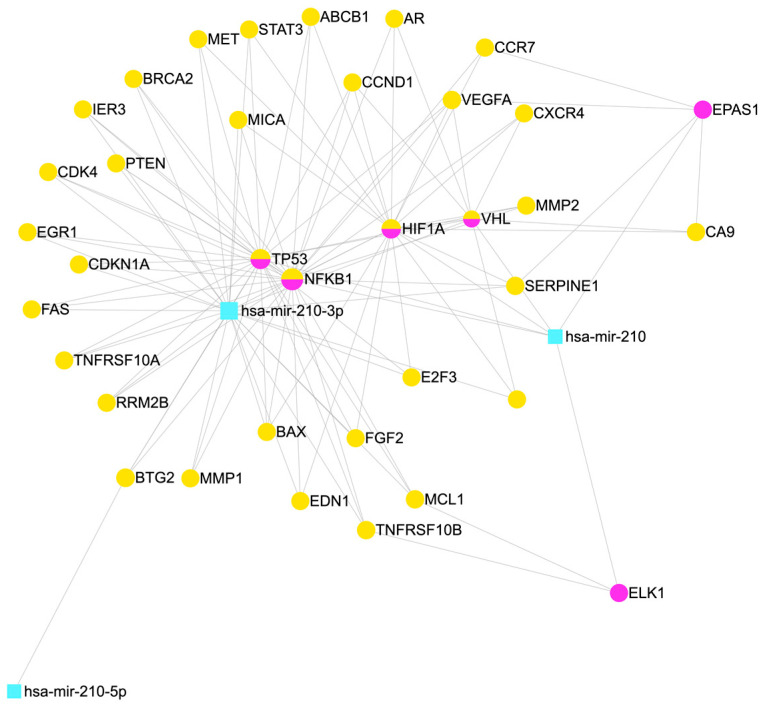
An interaction network to visualise the interaction of miR-210 and target genes. The pair-wise interaction is generated through miRNet (https://www.mirnet.ca/miRNet/home.xhtml) (accessed on 19 October 2023) [66]. Datasets are specialised to miRTarBase V8.0, miRecords, Transcription Factors. Organisms are specified as *H. sapiens* (human), and tissue type is not specified. Yellow nodes represent gene, pink nodes represent transcription factors, half yellow half pink are both genes and transcription factors while blue nodes used for miRNA. Edges represent the degree of interaction.

The currently identified downstream targets of miR-210 in PC are E2F3, EFNA3, GIT2, MNT, ZNF462, HOXA9, and EGR3 [43,56,61]. A miRNet interaction network was generated to visualize the miR-210 interaction with target transcription factors and genes [66] (Figure 3). Most of the downstream target genes are regulated through the HIF-1α-dependent pathway, even though HOXA9 is partially regulated through the HIF-1α-independent pathway [61].

E2F transcription factor-3 (E2F3) plays an oncogenic role in tumourigenesis, and changes in its function are associated with a poor prognosis in different types of cancers, highlighting its significance in clinical cancer outcomes [67]. Intermittent hypoxia (IH) is a distinguishing feature of obstructive sleep apnoea (OSA), which has been linked to tumour development and progression. In patients with lung adenocarcinoma and OSA, elevated levels of miR-210-3p were positively associated with polysomnographic parameters, including the oxygen desaturation index, apnoea–hypopnea index, and the proportion of total sleep time with oxygen saturation in arterial blood below 90%. IH augmented tumour viability, proliferation, migration, and invasion, led to the downregulation of E2F3 expression, and increased miR-210-3p levels. Mimicking this effect, overexpressing miR-210-3p induced similar alterations. These changes were rescued by inhibiting miR-210-3p in vitro or through the intra-tumoral injection of miR-210-3p antagomir in vivo [68]. In contrast, as mentioned above, it was reported that E2F3 is negatively regulated by miR-210, as upregulation of miR-210 leads to cell cycle arrest, reduced cell viability, and downregulates E2F3 expression in PC cells [65]. Similarly, another study presented compelling evidence implicating the HIF signalling pathway in the regulation of miR-210. This study confirmed a downregulation of E2F3 at the protein level following miR-210 induction [64]. Notably, a significantly high occurrence of miR-210 gene copy deletions was observed in ovarian cancer patients (64%, *n* = 114), and the finding established a correlation between gene copy number and miR-210 expression levels [69]. This controversy marks the need for future research and detailed elucidation of the HIF-1α-dependent regulatory pathway of miR-210 and E2F3 in PC.

Ephrin A3 (EFNA3), similar to many genes within the ephrin family, holds a pivotal role in embryonic development and is susceptible to dysregulation in various tumour types [70]. Recently, it was demonstrated that, under hypoxic treatment, mesenchymal stem cell-derived small extracellular vesicles (EVs) exert a notable impact on enhancing the proliferation, migration, and angiogenesis of human umbilical vein endothelial cells (HUVECs), along with facilitating vascularised bone formation [64]. More importantly, it was suggested that HIF-1α can trigger the upregulation of miR-210-3p in hypoxic conditions, leading to the inhibition of EFNA3 expression and subsequent activation of the PI3K/AKT pathway [71]. Another study delved into the roles of miR-210 in malignant peripheral nerve sheath tumour (MPNST) cells, revealing that elevated miR-210 levels heightened cellular viability, colony formation, the proportion of cells in the S phase, and invasiveness of MPNST cells [72]. Conversely, inhibiting miR-210 expression led to reduced proliferation and invasion of MPNST cells. These findings imply that miR-210’s promotion of proliferation and invasion, mediated through EFNA3, holds significance in MPNST tumourigenesis and progression [72]. Another study further elucidated the mechanism in PC, which indicates that miR-210 expression exhibits significant upregulation in both PDAC exosomes and malignant cells [73]. Elevated levels of miR-210 play a pivotal role in enhancing tumour angiogenesis, cell invasion, and proliferation in PDAC cells. Significantly, it was revealed that miR-210 exerts a negative regulatory effect on EFNA3 expression and actively participates in the PI3K/AKT/VEGFA or Wnt/β-catenin/RHOA pathways, thereby promoting tumour angiogenesis and cellular permeability. PDAC cells harness EV transmission of miR-210 to stimulate endothelial angiogenesis or permeability. In vivo studies confirmed that exosomal miR-210 drives PDAC progression [73].

MAX network transcriptional repressor (MNT) serves as a pivotal regulator of MYC, overseeing numerous cellular functions, and is known to be involved in most human cancers. MNT initially emerged as an antagonist of MYC and a tumour suppressor. Remarkably, approximately 10% of human tumours exhibit deletions in one MNT allele [74]. In gastric cancer, there is a notable increase in the expression levels of both miR-210 and its host long non-coding RNA, MIR210HG. Within this context, miR-210 exerts direct suppression of dopamine receptor D5 (DRD5), serine/threonine kinase 24 (STK24), and MNT. This suppression leads to heightened metastatic and invasive capabilities of GC cells. Notably, the MYC proto-oncogene (c-Myc) plays a pivotal role in transactivating both miR-210 and MIR210HG. Overexpression of miR-210 and MIR210HG, separately or jointly, can rescue the inhibitory effects on migration and invasion caused by c-Myc silencing. Furthermore, the administration of a c-Myc inhibitor significantly attenuates lung metastasis in GC in vivo [75]. However, the role of MNT/c-Myc/miR-210 in PC requires future research.

HOXA9 is a transcription factor involved in both the HIF-1α-dependent pathway and HIF-1α-independent pathway, in which the roles of HOXA9 in PC were newly identified. In the previous section, it is mentioned that the suppressive role of miR-210 on HOXA9 under hypoxic conditions influences the sensitivity of gemcitabine in PANC-1 cells [61]. Another relevant study regarding the role of HOXA9 under hypoxia and increased miR-210 in haemangioma might inspire research in hypoxia-induced PC-derived EVs [76]. Haemangioma (Hem) is a prevalent benign tumour frequently observed during infancy. This condition is influenced by HUVEC-derived EVs, which play an active role in Hem pathogenesis. Specifically, under hypoxic conditions, HUVECs induce the release of EVs, which are subsequently internalised by Hem endothelial cells (HemECs). These hypoxia-induced HUVEC-derived EVs promote HemEC proliferation and migration while inhibiting apoptosis. A notably high expression of miR-210 within these hypoxia-induced HUVEC-derived EVs contributes to the enhanced growth of HemECs through targeting HOXA9. Moreover, the inhibition of miR-210 expression in hypoxia-induced HUVEC-derived EVs demonstrates a suppressive effect on Hem development in vivo [76]. The detailed molecular mechanism of PC under hypoxic condition requires further elucidation.

### 3.3. Roles of miR-210 in PC Chemoresistance

The current therapeutic strategy for PC entails an initial surgical resection followed by adjuvant chemotherapy [77]. Over the past decade, gemcitabine has been rendered as the primary first-line treatment for advanced PC [78,79]. Despite the efficacy of gemcitabine and other chemotherapeutic agents in managing advanced and metastatic PC, the emergence of chemoresistance to gemcitabine presents an obstacle, substantially diminishing the therapeutic impact of this chemotherapy. It is noteworthy that PC cells exhibit a strengthened resistance to gemcitabine compared to other chemotherapeutic agents [80]. Given the shortage of research in this area, most investigations into chemoresistance in advanced PC have concentrated their efforts on gemcitabine [79]. Furthermore, exosomes originating from PC stem cells resistant to gemcitabine play a pivotal role in facilitating the horizontal transfer of drug-resistant characteristics to gemcitabine-sensitive PC cells through the exosomal delivery of miR-210 [63]. It was reported that miR-210 was significantly upregulated in gemcitabine-resistant PDAC cell lines carrying mutant p53 [81]. These discoveries suggest that targeted intervention of miR-210-contained EV is a potential therapeutic strategy for patients undergoing gemcitabine-based treatments. However, the precise molecular and cellular mechanisms underpinning miR-210 and the development of gemcitabine resistance in PC remain unclear.

### 3.4. miR-210 as a Potential Diagnostic Marker for Pancreatic Cancer Early Detection

It was suggested that miR-210 could be used as a diagnostic marker and prognostic factor for PC [82]. Another study explored the diagnostic potential of plasma miR-181b, miR-196a, and miR-210 in pancreatic cancer [83]. miR-210 is reliably detected and quantified, with a statistically significant 4-fold increase in expression observed in PC patients’ plasma samples (*n* = 40) compared to normal controls (*n* = 40, *p* < 0.00004) in the test dataset. This difference was subsequently confirmed in the validation group (*p* < 0.018), which was based on the quantification of CA199 [58,83]. Guz et al. (2021) suggested that miR-210-3p exhibits promising potential as a non-invasive serum biomarker for distinguishing between PDAC (*n* = 26) and chronic pancreatitis *(n* = 34, *p* = 0.015) patients [59]. Individuals with chronic pancreatitis face an elevated susceptibility to the development of PDAC. Through comparing the serum miR-10b-5p, and miR-210-3p expression profiles among patients diagnosed with chronic pancreatitis, PDAC, and a control group, it was shown that miR-210-3p revealed positive correlations with alkaline phosphatase and γ-glutamyltranspeptidase, which were cholestasis-related enzymes associated with PDAC. [59,84].

It was previously described that miR-210 expression is elevated in pancreatic juice and closely associated with lymph node metastasis, which represents a promising candidate biomarker for PDAC patients [85]. In an earlier study, miR profiling was conducted on exocrine pancreatic secretions, specifically pancreatic juice, utilising microarray analysis. The pancreatic juice samples were collected from six PDAC patients and two pooled samples from six individuals without pancreatic disease or other health issues. The enrichment of miR-210 in pancreatic juice was found to be associated with decreased overall survival (OS). Furthermore, higher levels of miR-210 were linked to the presence of lymph node metastasis [85]. However, another study reported a controversial result that elevated expression levels of miR-210 exhibited a noteworthy association with enhanced OS, which was statistically significant (*n* = 31, *p* = 0.003) [86]. To address the controversial results, future research needs to expand the sample size of PC and separately evaluate the OS among different stages of PC.

Nevertheless, aberrant expression of miR-210 is observed in various malignancies, including breast, prostate, colorectal, lung and ovarian cancers [87,88,89,90,91,92,93,94,95,96,97,98,99,100,101]. Several studies have demonstrated that colorectal cancer is associated with hypoxia caused by HIF-dependent upregulation of miR-210, while prostate cancer is correlated with hypoxia caused by HIF-independent upregulation of miR-210 [87,89,93,102,103,104,105,106,107]. These data suggest that the upregulation of miR-210 under hypoxic conditions is correlated with multiple malignancies, including PC, that fit cancer hallmarks. One of the challenges in the early detection and preventive screening of PC is the absence of sensitive and/or specific biomarkers [108]. Interestingly, analysing liquid biopsies from pancreatic juice might hint at a potential direction of utilising miR-210 as a PC circulating biomarker. PC exhibits a distinctive characteristic wherein tumour cells come into direct contact with both the exocrine and endocrine systems of the pancreas. Consequently, this proximity facilitates the release of diverse tumour-associated materials, including circulating tumour cells (CTCs), circulating tumour DNA (ctDNA), circulating tumour RNA (ctRNA), and EVs into pancreatic juice. This phenomenon renders pancreatic juice an optimal source for obtaining integrated liquid biopsies of PC [109,110]. It has been previously reported that serum-derived miR-210 carried in EVs is correlated with the disease progression in lung cancer, which indicates that profiling EV-associated miR-210 in pancreatic juice might be valuable to overcome the challenge in future research [101]. The stability of miR-21 that was carried by EVs from pancreatic juice versus circulating miR-21 was examined and it was found that the EV miR-21 is more stable [111].

## 4. Conclusions

In conclusion, hypoxia-induced upregulation of miR-210 plays a significant role in the complex landscape of PC. Hypoxia, a hallmark of PC, triggers the overexpression of miR-210 through HIF-dependent and HIF-independent pathways. miR-210 emerges as hypoxamir with multifaceted roles in PC. While the literature presents some conflicting findings regarding the precise role and expression of miR-210 in PC, it is evident that miR-210 influences critical cellular processes, including proliferation, angiogenesis, apoptosis, and metastasis. Its impact on downstream target genes like E2F3, EFNA3, GIT2, MNT, ZNF462, HOXA9, and EGR3 underscores its significance in shaping the aggressive phenotype of pancreatic cancer cells. Additionally, miR-210’s potential as a diagnostic marker for early PC detection is a promising avenue for further research.

## Figures and Tables

**Table 1:** The known targets and pathways of HIF-1α, HIF-2α, and HIF-3α.

HIFs	Target	Pathways
HIF-1α	NOS3, MCT4, CA-IX, PFK, LDHA, LDHA, GLUT1/3, HMOX1, BNIP3, IGF2, IGF-BP1, IGF-BP3, c-Myc, VEGFA, GLUT1, EPO	p53, Angiogenesis, Adipogenesis, AP-1 transcription factor network, Survival, Proliferation
HIF-2α	VEGFA, GLUT1, EPO, MMP2, MMP13, OCT-3/4	p53, Angiogenesis, Adipogenesis, AP-1 transcription factor network, Survival, HIF-1α transcription factor network
HIF-3α	Inhibits HIF-1α and HIF-2α	PI3K/Akt/mTOR, Hypoxic and oxygen homeostasis by HIF-1α

## Data Availability

Data contained within the article.
